# Feasibility, acceptability, and potential efficacy of an innovative postnatal home-based breastfeeding peer support programme in Hong Kong: a feasibility and pilot randomised controlled trial

**DOI:** 10.1186/s13006-021-00381-5

**Published:** 2021-04-13

**Authors:** Kris Yuet-Wan Lok, Charlotte L. Y. Chow, Jeffery Sheung Yu Shing, Robert Smith, Christine Chi Oi Lam, Debra Bick, Yan-Shing Chang

**Affiliations:** 1 School of Nursing, Li Ka Shing Faculty of Medicine, The University of Hong Kong, 4/F, William MW Mong Block, 21 Sassoon Road, Pokfulam, Hong Kong; 2 Department of Obstetrics & Gynaecology, Queen Elizabeth Hospital, Kowloon, Hong Kong; 3 Warwick Clinical Trials Unit, Warwick Medical School, University of Warwick, Warwick, Gibbet Hill CV4 7AL UK; 4 Florence Nightingale Faculty of Nursing, Midwifery & Palliative Care, King’s College London, James Clerk Maxwell Building, 57 Waterloo Road, London, SE1 8WA UK

**Keywords:** Peer support, Breastfeeding, Pilot

## Abstract

**Background:**

As suggested by the World Health Organization, breastfeeding peer support is being introduced worldwide to support women’s breastfeeding needs. Evidence has shown that when such support is offered to women, the duration and exclusivity of breastfeeding is increased. We developed an innovative home-based intervention to sustain exclusive breastfeeding in Hong Kong. However, potential barriers must be addressed before a full randomised controlled trial (RCT) is conducted. The aim of this study was to determine the feasibility of a breastfeeding support programme with home-based visits from peer supporters over a six month period among postpartum Chinese women in Hong Kong.

**Methods:**

We conducted a feasibility and pilot randomised controlled trial. Twenty primiparous women intending to breastfeed their healthy term singleton infant were recruited from a hospital in Kowloon, Hong Kong between February and March 2019. Participants were randomly allocated to the intervention or control group. Participants in the intervention group received five home-based visits with a peer supporter over a six month period, as well as standard care, whereas participants in the control group received standard care only. We assessed feasibility, compliance, and acceptability of the breastfeeding peer support programme. Other outcomes assessed were breastfeeding self-efficacy, duration, and exclusivity.

**Results:**

It was feasible to recruit and train existing peer supporters, and peer supporters were able to deliver the intervention, which was acceptable to women, but rates of stopping the intervention and loss to follow-up were high. There was higher retention seen within the first month. Women interviewed at the end of the study reported that the intervention was positive. The cessation risk of any, and exclusive breastfeeding were not statistically different between the intervention and control groups.

**Conclusions:**

This study provided valuable information on feasibility of the trial design and intervention. Modifications to the intervention, such as targeting women with lower breastfeeding self-efficacy, or combining home visits with technology and telephone follow-up may be more appropriate in a larger trial. Implementing the programme early during the antenatal phase and tailoring peer support to most appropriately sustain exclusive breastfeeding and other feeding modes should be incorporated in a future home-based peer support arm.

**Trial registration:**

NCT03705494 on 15 Oct 2018.

## Background

The benefits of breastfeeding for women and their children have been well evidenced in the past decade [1, 2], and the economic costs associated with infants who are not breastfed has been quantified [2]. The World Health Organization (WHO) has set a target to increase exclusive breastfeeding rates globally during the first six months postpartum to 50% by 2025 [3]. Hong Kong has high breastfeeding initiation, with more than 88% of women initiating breastfeeding; however, only half of these women will exclusively breastfeed their babies [4]. Despite the importance of exclusive breastfeeding for the first six months postpartum, the maintenance of exclusive breastfeeding remains a major public health issue. This is particularly true in Hong Kong where there is a high drop off rate in exclusive breastfeeding during the first two months following birth and low proportion of infants exclusively breastfed for the first 4–6 months [4].

Early breastfeeding cessation in developed countries has been attributed to a wide range of factors such as sociodemographic characteristics, maternal employment, hospital practices, maternal confidence, family infant feeding preferences, and breastfeeding support [5, 6]. Breastfeeding support is a modifiable factor that has been extensively investigated to assess its association with breastfeeding. In Hong Kong, standard antenatal and postpartum care consists of routine antenatal and perinatal care, with group or one-on-one antenatal and postnatal lactation education provided by a midwife or lactation consultant, one-on-one assistance with breastfeeding if problems arise, and post-discharge follow-up either at an outpatient clinic in the maternity hospital or nearest Maternal and Child Health Centre. However, the most recent survey showed that the exclusive breastfeeding rate at six months in Hong Kong, as determined using the WHO guidelines and since birth, was 26.3% [4]. Strategies to support breastfeeding maintenance are needed, with a focus on cultural needs and on groups who are less likely to initiate and continue breastfeeding. Breastfeeding peer support groups are available in community settings, but are not practical for Chinese women who practise the tradition of ‘doing the month’ in which women remain housebound during the first month post-birth. Thus, women who are usually housebound during the early phase of the postnatal period may be better served by home visits. Evidence from a Cochrane review suggested that provision of postnatal care at home could reduce utilisation of infant health services and higher frequencies of postnatal home visits could encourage more women to exclusively breastfeed [7]. In developed countries, healthy women and babies are usually discharged from the hospital within 1–2 days after birth [7]. In Hong Kong, the average length of inpatient stay for uncomplicated vaginal birth is around 48 h and 72 h for cesarean delivery [8]. Home visits during the first few days after birth by healthcare professionals or trained support workers could offer opportunities to assess the woman and newborn and provide health education, infant feeding support, and emotional and practical support.

As suggested in the WHO strategy, breastfeeding peer support is being introduced worldwide to support women’s breastfeeding needs [2]. Dennis (2003) considered peer support to be ‘assistance by a created social network member’, which extends embedded social networks and complements healthcare professional services [9]. A Cochrane review of support for breastfeeding women, which aimed to investigate the kinds of support that can help women solve breastfeeding problems, found that trained volunteers and doctors and nurses have a positive impact on breastfeeding and that face-to-face support is associated with better outcomes than telephone-only support [10]. When breastfeeding support is offered to women, the duration and exclusivity of breastfeeding is increased. Characteristics of breastfeeding support that have been shown to be most effective include face-to-face contact and volunteer support, which incorporate ongoing scheduled visits so that women can anticipate when support will be available [10]. Another systematic review of randomised controlled trials (RCTs) indicated that community-based peer support for women was effective in increasing the duration of exclusive breastfeeding. Moreover, women were more likely to exclusively breastfeed when they received peer support one on one or through a support group, as compared with women who had not received such support [11]. Another systematic review also found that peer support aimed at increasing continuation of breastfeeding was more effective with five or more planned peer-to-mother contacts and when provided during the postnatal period [12]. Several more recent studies have also found positive effects on infant feeding outcomes using a proactive community asset-based approach underpinned by behaviour-change techniques, such as receiving proactive contact from infant feeding helpers via face-to-face contact or regular text-based communication both during pregnancy and in the first few weeks after birth; these findings warrant further research [13, 14]. A more recent large RCT yielded evidence that providing first-time mothers with proactive telephone-based peer support increased any breastfeeding at six months; however, no effect was found on exclusive breastfeeding duration [15]. The inconsistent results for the different support delivery modes highlight the importance of culturally specific interventions for different populations.

Currently, a range of community or telephone-based breastfeeding peer support programmes is available in Hong Kong [16, 17]. The effectiveness of home-based peer support during the early postpartum period have not been investigated. In cultures such as that of Hong Kong, home-based visits may be a potentially beneficial approach to incorporating peer-support without breaking with cultural traditions.

We developed an innovative home-based intervention to promote and sustain exclusive breastfeeding, with the aim to address an important service gap in Hong Kong. However, there might be potential barriers to successful implementation and to evaluating the effectiveness of the intervention. This is the first postnatal breastfeeding support programme based on home visits provided by peer supporters in Hong Kong. Before proceeding with a full-scale RCT, a feasibility and pilot study was needed to identify whether a home-based breastfeeding peer support programme is feasible and acceptable. The main purpose of the current study was to determine the feasibility of conducting a breastfeeding support programme with home-based visits from peer supporters over a six month period among postpartum Chinese women in Hong Kong.

## Methods

### Participant recruitment, randomisation, and blinding

This study targeted healthy mother–infant pairs and primiparous women who intended to breastfeed. All eligible women were approached by a research assistant on a postnatal ward until the target sample size (20 eligible participants) was met. The inclusion and exclusion criteria are summarised in Table 1.

**Table 1:** Inclusion and exclusion criteria of participants in the study

Inclusion criteria	Primiparous woman
	Intending to breastfeed (any)
	Singleton pregnancy
	Term infant (37–42 gestational weeks)
	Speaks Cantonese
	Hong Kong resident
	No serious medical or obstetrical complications
**Exclusion criteria**	Infant with Apgar score < 8 at 5 min
	Infant with birthweight < 2500 g
	Infant with any severe medical condition or congenital malformation
	Infant was placed in the special care baby unit for more than 48 h after birth
	Infant was placed in the neonatal intensive care unit at any time after birth

Our target sample size was 20 participants (10 per arm), based on the aims of this feasibility and pilot study; that is, to gauge the rate of recruitment, adherence, and retention levels, as well as to identify any unanticipated issues that would need to be resolved prior to conducting a full-scale randomised control trial. This is also the recommended sample size for a feasiblity and pilot study where a full-scale trial would have an anticipated medium effect size (Cohen’s *d* = 0.50) with a power of 0.80 [18].

Eligible participants who provided consent to participate were randomly assigned (1:1) using random block lengths of 2, 4, and 6, into either the control group (usual care) or the intervention group (home-based breastfeeding peer support programme together with usual care). The group randomisation sequence was generated by a computer using Stata version 16 (9) and held by an independent researcher outside the research team who did not participate in participant recruitment, data collection, or analysis. Three different individuals are involved to maintain allocation concealment. The independent researcher informed the practice nurse at the postnatal ward of allocation via telephone, after the study’s research assistant completed baseline participant data collection of demographic information and other study measures at study entry. The study’s research assistant remained blinded to allocation during the whole study. The study was conducted and reported following the CONSORT (Consolidated Standards of Reporting Trials) guidelines.

### Intervention

The intervention was based on a community “Breastfeeding Peer Support” scheme organised by the non-government organisation (NGO) Natural Parenting Network and the Department of Health, adapted to incorporate home-based-specific elements [19]. Peer supporters were adult Cantonese-speaking women recruited by the Natural Parenting Network. This NGO has previously been commissioned by the Department of Health in Hong Kong to train breastfeeing peer supporters, and over 100 volunteers have been trained. The volunteers were recruited from their existing network of volunteers. Peer supporters in the network are all volunteers, but their travel expenses for home visits are reimbursed. The lead peer supporters are qualified after receiving at least 16 h of training under the “Baby Angel Friendly Scheme” and after passing a standardised assessment by their trainers. Eligibilty criteria for peer supporters in this study were having had at least four months breastfeeding experience themselves. The training for peer supporters using the Baby Angel Friendly Scheme was provided by the lead peer supporter from Natural Parenting Network and covered topics such as (1) why breastfeeding is important, (2) how to ensure a good start to breastfeeding, (3) how to help mothers breastfeed (4) communication skills, (5) common breastfeeding problems, (6) diet and hygiene, (7) local support, and (8) role of peer supporters. Home-based-specific training was provided by a lactation consultant midwife on issues that peer supporters may encounter during home-based visits such as peer supporter safety, handling emergency situations, and supporting breastfeeding. There was no explicit exclusion of health professionals, but all peer supporters were volunteers with experiential knowledge. Peer supporters were to refer mothers to health professionals if there were specific breastfeeding problems that peer supporters were unable to help with.

The home-based breastfeeding peer support programme comprises five home visits, based on a systematic review suggesting that interventions are more effective with five or more planned peer-to-mother contacts during the postnatal period [12]. Two visits were conducted during the first month postpartum, with the first visit made within the first week postpartum and the second within the third week postpartum. Thereafter, three subsequent visits were conducted at 2, 4, and 6 months postpartum. The first home visit was conducted during the first week postpartum to provide timely and early breastfeeding support, and subsequent visits were timed to provide continuous support for breastfeeding mothers at different stages. We considered that providing early support within the first week postpartum would be helpful in resolving early breastfeeding difficulties and providing mothers with timely support. Each visit lasts for 30 min to an hour. Peer supporters established phone contact with participants within the first 48 h postpartum to schedule the first visit and subsequent visits. Between visits, WhatsApp and telephone support were proactively initiated by the peer supporter to the participant or vice versa when needed. Peer support visits are terminated when participants decide to stop breastfeeding.

During the study period, the control and intervention groups received the standard antenatal and postpartum care given in Hong Kong. The control group did not receive home-based breastfeeding peer support.

### Data collection and outcomes

The primary study outcomes were to identify whether it was feasible to recruit women, randomise participants, and follow up participants until six months postpartum, as well as explore whether the home-based peer support programme was acceptable to participants. Secondary outcomes were the potential efficiacy of the breastfeeding peer support programme.

Three questionnaires were designed to collect relevant data during the immediate postpartum period. One survey was completed by participants regarding sociodemographic characteristics and breastfeeding self-efficacy. Two data extraction forms were used to collect maternal birth data and infant feeding data, which were retrieved from the medical records with participant’s consent by the research team. Telephone follow-up was conducted at one, two, four, and six months postpartum or until participants stopped breastfeeding, which ever came first, to collect data on infant feeding status (detailed below). Data of breastfeeding self-efficacy (see below) were also collected via telephone follow-up at two months. A research assistant collected all baseline and follow-up outcome data while remaining blinded to participant allocation. Views on the intervention and how it can be improved were sought from participants through in-depth qualitative interviews on completion of the study.

#### Infant feeding status

This was measured using the existing WHO categories and defined as since birth [3]. Infants were considered exclusively breastfed if they received no solids, no other breast milk substitutes, and no water or other liquids (other than vitamins or medications) [3].

#### Breastfeeding self-efficacy

Maternal breastfeeding self-efficacy was measured at baseline and at 2-month follow-up using the Breastfeeding Self-Efficacy Scale Short-Form (BSES-SF) (Chinese version) [20]. The BSES-SF consists of 14 items measured on a five-point Likert scale that begins with the phrase “I can always. ..” On the scale, 1 indicates “not at all confident” and 5 indicates “always confident”. Total scale scores range from 14 to 70, with higher scores indicting greater breastfeeding confidence and self-efficacy [20].

### Statistical analysis

Descriptive analysis was used to describe feasibility of recruitment, retention, and study participants’ characteristics. To compare baseline characteristics between the two study groups, a chi-square test was used for categorical variables. To test the potential efficacy of the intervention, the primary analysis was an intention-to-treat analysis that compared all participants’ time to stopping exclusive breastfeeding from baseline to either one, two, four, or six months postpartum using a Cox proportional hazards model, taking the controls as the reference group. The proportional hazard assumption was tested by examining for symmetry on a log-log survival plot by study group and non-significant *p* - values in the Schoenfeld residuals test [21]. We used a Kaplan–Meier survival curve in relation to study group at the start of randomisation until six months postpartum [22]. As a secondary analysis, the same model as in the primary analysis was used to assess all participants, the maintenance of any and exclusive breastfeeding from the start of randomisation until six months postpartum. We conducted an intention-to-treat analysis using a linear regression model to examine the differences in BSES-SF scores between groups. The linear regression model examined the difference between study groups at two months postpartum while adjusting for baseline BSES-SF scores, taking controls as the reference group. Where appropriate, each estimate was accompanied by a 95% confidence interval (CI), and a 5% level of significance was used in all statistical tests except where specified. The analysis was performed using Stata version 16.0 statistical software [23], and the statistician was blinded to allocation when conducting the analysis.

### Qualitative data analysis

All women in the intervention group who received the intervention and not lost to follow-up were approached for the interview at study completion. Eight participants had received intervention, of which two had been lost to follow-up at that point and one has refused to be interviewed. Thus, five participants were interviewed. The interview was conducted by the principal investigator, and transcription of each interview was completed by research assistants. Translation and cross-checking were completed by two bilingual research assistants fluent in both Cantonese and English. We used a two-step thematic analysis process [24]. First, the research team read and re-read each transcript and then developed an open code list derived directly from the data, to provide a greater opportunity for participants’ voices to drive the analysis. All relevant textual data were coded. The second level of analysis involved grouping the codes thematically using a process of contextualizing codes into conceptually similar and overarching themes [24]. We used a manual data management strategy for analysing small scale qualitative data, as laid out by Webb, to map out broad categories of information as such a strategy provides the researcher greater familiarity and intimacy with the participants’ belief and perceptions [25] when there are fewer than 10 study participants interviewed. Themes were generated and the theme names established; then, the meanings were grasped and formulated, and hidden meanings and contextual factors were explored.

Ethical approval was obtained from the Institutional Review Board of the University of Hong Kong/Hong Kong West Cluster. Informed written consent was obtained from all participants. This trial is registered in ClinicalTrials.gov (NCT03705494, 15 Oct 2018).

## Results

### Feasibility of recruitment

**Fig. 1:**
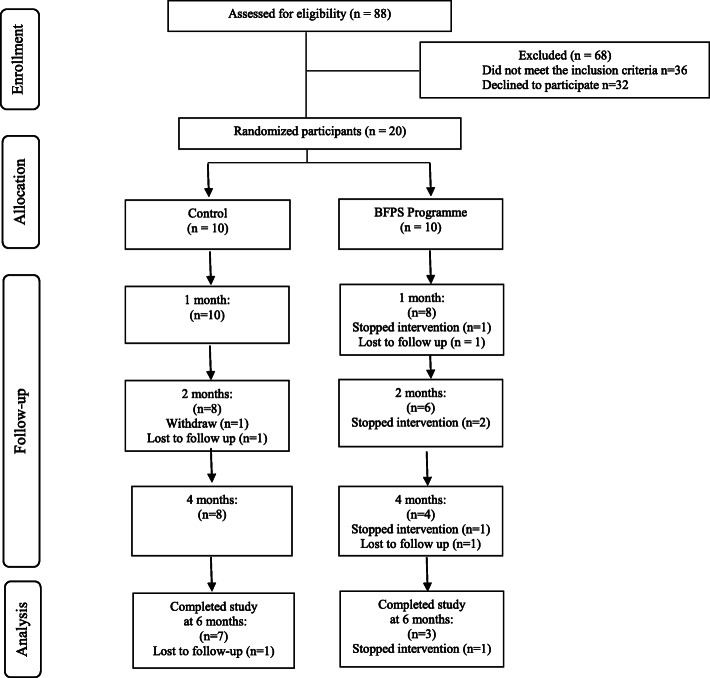
Participant flow diagram according to Consolidated Standards of Reporting Trials (CONSORT)

Participant recruitment began in February 2019; the final participant was randomised in March 2019, and the final follow-up outcome measurement completed in September 2019. A total of 88 women from postnatal wards were screened for eligibility, among whom 36 did not meet the inclusion criteria. In total, 38% (*n* = 20/52) of the eligible women approached agreed to participate in the study and were randomised into two arms: 10 to the control group and 10 to the intervention group. Of the 20 participants allocated, seven stopped the intervention and three were lost to follow up (Fig. 1). Reasons for stopping the intervention included the husband disagreeing with home visits, weaning and thus stopping home visits, and participants feeling pressured to breastfeed from peer supporters.

### Baseline characteristics

There were no statistically significant differences between groups in demographic characteristics, family preference regarding breastfeeding, or other study measures at baseline (Table 2). Most women in both groups planned to exclusively breastfeed: 90% in the intervention group (*n* = 9/10) and 80% in the control group (*n* = 8/10). Of those who planned to exclusively breastfeed, eight participants in the control group and seven in the intervention intended to exclusively breastfeed for six months. Seventy percent (*n* = 7/10) of mothers in the intervention group said they would return to work postpartum whereas 90% (*n* = 9/10) of controls said they would do so. On average, participants returned to work after 11.4 weeks, which is similar to the 10-week standard maternity leave in Hong Kong at the time of the study before it was increased to 14 weeks.

**Table 2:** Comparison of characteristics of participants by two groups, (*n* = 20)

Characteristics	Intervention arm N (%) ***N*** = 10	Control arm N (%) ***N*** = 10	***p***-value
Maternal age			0.308
18–24 years	3 (30.0)	0 (0)	
25–29 years	1 (10.0)	1 (10.0)	
30–34 years	4 (40.0)	6 (60.0)	
≥ 35 years	2 (20.0)	3 (30.0)	
Maternal education			0.528
Primary	0 (0)	0 (0)	
Compulsory Secondary	0 (0)	1 (10.0)	
Post-Secondary	5 (50.0)	4 (40.0)	
University Degree	4 (40.0)	5 (50.0)	
Post-Graduate Degree	1 (10.0)	0 (0)	
Monthly family income (Hong Kong Dollar)			0.819
< HK$15,000	1 (10.0)	1 (10.0)	
HK$15,000-HK$29,999	2 (20.0)	1 (10.0)	
≥ HK$30,000	7 (70.0)	8 (80.0)	
Length of Residence in Hong Kong			0.305
< 5 years	0 (0)	1 (10.0)	
5 to < 10 years	0 (0)	0 (0)	
10 to < 15 years	0 (0)	0 (0)	
≥ 15 years	10 (100.0)	9 (90.0)	
Returning to work postpartum			0.472
No or not sure	3 (30.0)	1 (10.0)	
Yes – within 10 weeks postpartum	4 (40.0)	4 (40.0)	
Yes – longer than 10 weeks postpartum	3 (30.0)	5 (50.0)	
Parity			0.531
Primiparous	9 (90.0)	8 (80.0)	
Multiparous	1 (10.0)	2 (20.0)	
Delivery type			0.133
Spontaneous vaginal	3 (30.0)	7 (70.0)	
Assisted vaginal	4 (40.0)	0 (0)	
Planned caesarean	2 (20.0)	2 (20.0)	
Emergency caesarean	1 (10.0)	1 (10.0)	
Self-breastfed			0.371
No	6 (60.0)	4 (40.0)	
Yes	4 (40.0)	6 (60.0)	
Attended antenatal childbirth class			1.000
No	2 (20.0)	2 (20.0)	
Yes	8 (80.0)	8 (80.0)	
Attended antenatal breastfeeding class			1.000
No	3 (30.0)	3 (30.0)	
Yes	7 (70.0)	7 (70.0)	
Partner’s Infant feeding preference			1.000
Breastfeeding	4 (40.0)	4 (40.0)	
Infant Formula	0 (0)	0 (0)	
Mixed Feeding	0 (0)	0 (0)	
No Preference	6 (60.0)	6 (60.0)	
Maternal mother’s Infant feeding preference			0.549
Breastfeeding	3 (30.0)	2 (20.0)	
Infant Formula	0 (0)	1 (10.0)	
Mixed Feeding	0 (0)	0 (0)	
No Preference	7 (70.0)	7 (70.0)	
Paternal mother’s Infant feeding preference			0.559
Breastfeeding	4 (40.0)	5 (55.6)	
Infant Formula	1 (10.0)	0 (0)	
Mixed Feeding	0 (0)	0 (0)	
No Preference	5 (50.0)	4 (44.4)	
When decision to breastfeed was made			0.350
Before becoming pregnant	1 (10.0)	3 (30.0)	
During 1st trimester	8 (80.0)	6 (60.0)	
During 2nd trimester	0 (0)	0 (0)	
During 3rd trimester	0 (0)	1 (10.0)	
After baby born	1 (10.0)	0 (0)	
Planned to exclusively breastfeed			0.301
No	1 (10.0)	2 (20.0)	
Yes – for 6 months	7 (70.0)	8 (80.0)	
Yes – for less than 6 months	2 (20.0)	0 (0.0)	
Planned duration of any breastfeeding			0.305
< 6 months	1 (10.0)	0 (0)	
≥ 6 months	9 (90.0)	10 (100.0)	

### Acceptability of the intervention

Half (*n* = 5/10) of participants randomised to the intervention group stopped the intervention. Of these, one participant stopped during the first month and did not complete all follow-up data collection. The other four participants stopped receiving the intervention at 2, 4, or 6 months but had completed all follow-up outcome measurements. The mean number of intervention sessions was 2.2 (standard deviation [SD]: 1.48), *n* = 10, with 30% (*n* = 3/10) completing the intervention dose of four sessions. Sixty percent (*n* = 6/10) had completed the intervention at 2 months, 40% (*n* = 4/10) at 4 months, and 30% (*n* = 3/10) at 6 months. The intervention was delivered face to face. In the qualitative interview with five mothers in the intervention group, participants expressed that home-based visits were convenient and provided them with useful breastfeeding information and emotional support. Participants suggested improvements for the intervention, including more frequent home visits at the beginning and greater complementary feeding support toward six months postpartum.

### Infant feeding outcomes

**Fig. 2:**
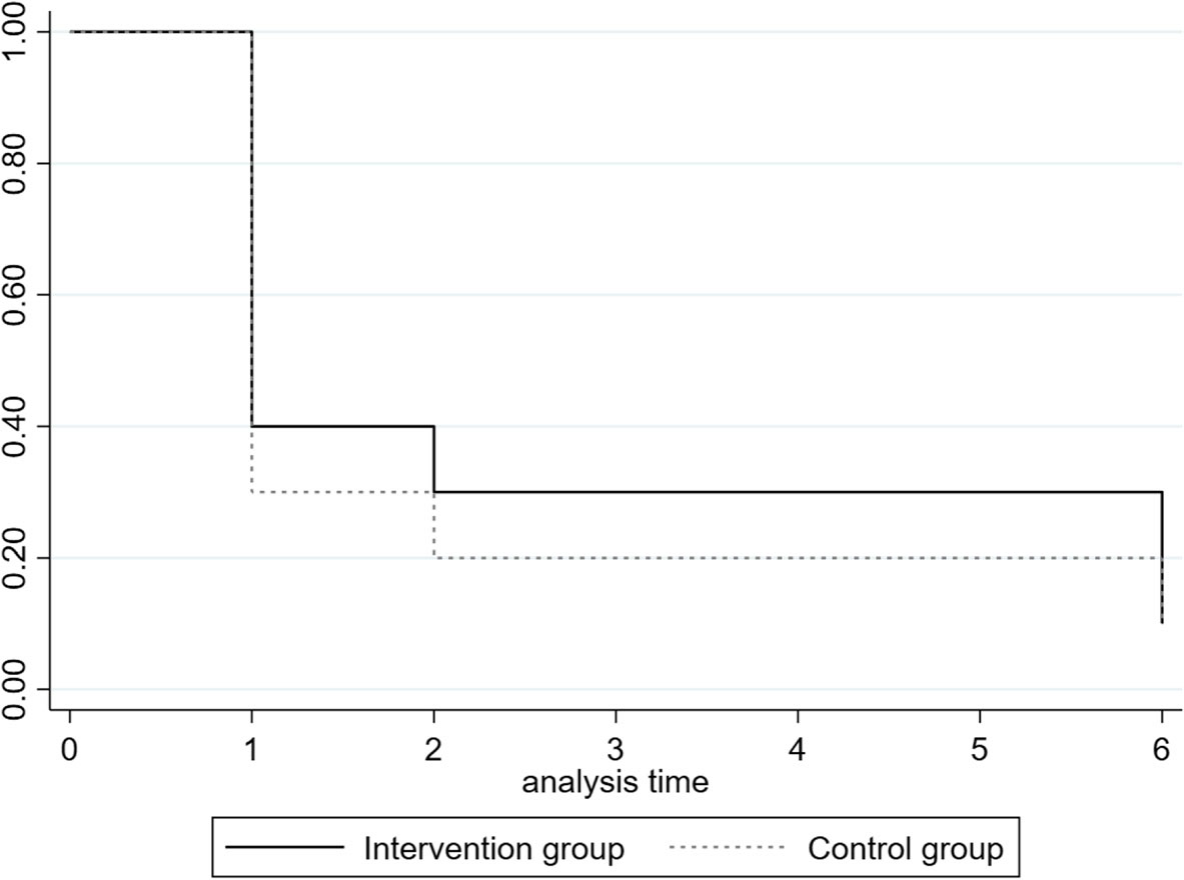
Kaplan-Meier curve of time to stopping exclusive breastfeeding between intervention and control group

At one month postpartum, all participants reported that they were exclusively breastfeeding. Among those who initiated breastfeeding in the intervention group, two women stopped exclusive breastfeeding at three days and before the first intervention (conducted during the first week) and continued any breastfeeding for 8.7 and 24 weeks. We collected data on the time when women stopped exclusively breastfeeding, based on recall. Four women stopped breastfeeding within the first week, three of whom were in the control group. The remaining women who initiated exclusive breastfeeding continued until at least four weeks (i.e., one month). This indicated that the current timing of the first visit during the first week should be retained, with possible consideration to include first contact via phone earlier to provide timely support where needed. According to the intention-to-treat analysis of all 20 randomised participants, one participant (10%) from each group was exclusively breastfeeding at six months. The log-log survival plot was symmetrical between groups and the Schoenfeld residuals test showed no statistically significant difference, suggesting that the assumption of proportional hazards was met (*X*^2^ = 0.11 [*df* = 1], *p* = 0.74). Over the entire six month postpartum period, no statistically significant difference was found between the intervention and control groups for any breastfeeding (hazard ratio [HR] = 0.36; CI 0.08–1.37, *p* = 0.128) and maintaining exclusive breastfeeding (HR = 1.48; CI 0.52–4.22, *p* = 0.46) (Fig. 2).

In terms of breastfeeding self-efficacy, no statistically significant differences were found between the intervention and control groups at 2-month follow-up after adjusting for baseline differences (*β* = 0.48, standard error [SE] = 7.98, *t* (11) = 0.6, *p* = 0.95), with the model showing low goodness-of-fit (R^2^ = 0.15, F [2, 9] = 0.82, *p* = 0.47), as shown in Table 3.

**Table 3:** Comparison of BSES of participants at baseline and at 2 months by two arm, (n = 20)

Mean BSES score (SD)	Intervention arm N (%) N = 10	Control arm N (%) N = 10	Difference between groups mean (95%CI)
Baseline	37.10 (10.80)	44.56 (6.21)	−7.46 (−16.12, 1.21)
2 months postpartum	45.17 (13.96)	45.00 (13.37)	0.17 (−16.54, 16.87)

### Positive aspects of breastfeeding peer support

Six women who received the intervention and were not lost to follow up were invited to be interviewed regarding the programme after six months postpartum. Five out of six mothers invited, including those who stopped the intervention, agreed to be interviewed. Most respondents felt that the primary benefits of the peer support programme were convenience and providing advice and encouragement that was readily available. Most importantly, peer supporters acted as a source of knowledge and emotional support for participants.

*“I can always talk to the volunteer about my questions, such as about breastmilk, because sometimes it is not possible to go to the Maternal Child Health Clinic and talk to a nurse immediately. .. [Volunteers are] experienced mothers that share experiences and answer my questions immediately.”*

*(M1)*

*“My failures in breastfeeding made me feel very frustrated and I had postpartum depression at that time, so I kept crying when [the volunteers] came to my home the first two times. They gave me emotional support.” (M4).*

### Confidence in breastfeeding

The interview data showed that most interviewees found that peer supporters were able to provide psychological support, which helped them feel more confident with breastfeeding.

*“Sometimes you need to understand from a psychological point of view, not just teach how to feed and massage. The psychological problems are more important than the positioning.” (M3).*

### Suggestions to improve peer support programme

Most respondents reported that the programme met their expectations and they got along with the peer supporters. It was suggested that peer supporters should be introduced early, to help prepare mothers for breastfeeding during pregnancy rather than after birth. Incorporating the peer support programme into childbirth classes during pregnancy was also recommended.

*“In addition, I was very tired because a lot of things were decided or learned after giving birth, I felt I couldn’t handle it mentally at the time. If I were well prepared before birth, it would be much better than preparing after birth.” (M4).*

Most peer supporters communicated through WhatsApp in addition to home visits, so using a mobile app can increase the contact frequency if face-to-face contact is not possible. Some participants preferred more contact with peer supporters via digital technologies, which may be a good option for the sustainability and cost reduction of future programmes.

*“. .. such as FaceTime, which is convenient for volunteers because they don’t have to do home visits, and also good for mothers; otherwise, the mothers have to arrange a time to meet [volunteers], which will be stressful.” (M1).*

## Discussion

The findings of this study demonstrated that it was feasible to recruit and train existing peer supporters, and peer supporters were able to deliver the intervention, which was acceptable to women. In addition, the current programme could be financially sustainable as the peer supporters were trained under the Department of Health’s Baby Angel Friendly Scheme, which has trained over 100 volunteers. Thus, the cost of training in this study was minimal. The peer supporters in this programme will train future peer supporters and all peer supporters are volunteers. Therefore, further research is needed to test the sustainability and cost-effectiveness of the scheme for training peer supporters to provide home-based breastfeeding peer support.

The challenges experienced could involve the fact that recruitment in postnatal wards may not be appropriate as women have just given birth, with approximately 38.5% of eligible women agreeing to participate in this study. Therefore, for a full-scale RCT, recruitment during the antenatal period should be considered, to increase the number of women who agree to participate in a trial. For those who participated in the intervention, the rates of stopping the intervention and loss to follow-up were high. We did not record all the reasons for stopping. However, in a similar study conducted in Hong Kong by Wong and colleagues in 2007, with a telephone peer counsellor breastfeeding intervention, 200 of 368 mothers approached participated in the study [26]; the uptake was approximately 54.3%, which was slightly higher than the rate in the current study. However, our study involved home visits rather than telephone interviews, suggesting home visits combining technology and telephone follow up can be further explored in a larger RCT design.

Although a large number of women stopped the intervention (50%), the number lost to follow-up was similar in the two groups. This showed that at least half of eligible women benefited from and agreed with the intervention. The remaining half of participants may have had cultural issues with home visits that involved family members. The challenges experienced indicate that for the full RCT, we may need to involve and obtain consent from both parents. The challenges experienced can inform the recruitment method used in the full RCT, to successfully increase recruitment and retention. Additionally, the rates of recruitment and retention will inform the sample size calculation in a future full-scale RCT.

From previous studies on peer support, women-centred rather than breastfeeding-focused support may improve acceptability to women [27, 28]. While the aim of the peer support is to improve the exclusive breastfeeding rate, it is important to focus the support to help women in achieving their breastfeeding goals rather than putting unnecessary pressure on them. In cultures such as that of Hong Kong where mixed feeding is common, peer support for women who eventually decided to adopt mixed feeding may be important to reduce the risk of isolating women and improve the reach and retention of the intervention [28, 29]. We have learnt from the high rates of stopping the intervention and loss to follow-up that we need to consider further training for peer supporters with respect to working with mothers who decide not to exclusively breastfeed, but may still need support in breastfeeding.

Higher retention was seen among new mothers within the first month, when help was most needed to establish breastfeeding, irrespective of their intended duration of breastfeeding. Therefore, a more flexible approach to the number of visits and modification of the intensity of intervention should be considered in the full-scale RCT study.

Women who remained in the intervention generally shared favourable comments and were highly satisfied with the programme, acknowledging the peer supporter as a helpful source of information and support; this finding is consistent with previous studies [11, 30]. The home-visiting aspect was also well received owing to its convenience for women during the postpartum period. However, participants suggested the use of digital technologies as an alternative to in-person visits. This is consistent with the qualitative literature in that access to timely support is an important component of breastfeeding outcomes [31].

With respect to potential efficacy of the intervention, when compared with the control arm, the intervention arm showed a lower risk of weaning from exclusive breastfeeding, although this was not statistically significant. However, because this study was not powered to detect significant differences between groups and the study’s purpose was to identify its feasibility and acceptability, this finding might be indicative of efficacy but is not conclusive, warranting further investigation. Nevertheless, the qualitative findings showed enriching benefits of the peer support programme in providing postnatal mothers with psychological support that helped their perceived confidence in breastfeeding.

This study used the BSES-SF to examine the changes associated with breastfeeding confidence related to time and the help of peer supporters. We observed a trend in increased breastfeeding self-efficacy in both the intervention and control arms, although no statistically significant difference was seen. It was expected that women would feel more confident and comfortable with breastfeeding over time because this change has been reported previously [32, 33]. The peer support intervention had a greater impact on self-efficacy, comparing baseline to two month follow-up between groups, although no significant difference was found. In this study, despite being positively perceived by participants in the intervention group, the value added by home-based peer support did not translate into higher breastfeeding self-efficacy when compared with current postnatal and community care. In the full RCT, we may need to target women who have lower breastfeeding self-efficacy and who would most benefit from this intervention.

Overall, the findings from this study suggest that it is feasible for peer supporters to deliver the intervention. However, modifications to the recruitment methods and a more flexible approach to the number of visits, as well as modification of the intensity of the intervention, would be needed to increase recruitment and retention. Additionally, to reduce the risk of isolating women and improve reach and retention of the intervention, a women-centred component of the intervention should be included in peer support, as non-exclusive breastfeeding may be important. Home visits by a peer support volunteer to provide support for breastfeeding was viewed positively by those who remained in the intervention. Participants expressed that the strength of the intervention was enabling them to discuss breastfeeding at home, which was preferable than in a clinic; however, the effectiveness warrants further study. A potential limitation is that the study relied on maternal self-reports of breastfeeding outcomes as it may contain recall bias. However, maternal recall has been found to be a valid and reliable estimate of breastfeeding duration [34]. Additionally, the study participants were a homogenous group of breastfeeding primiparous mothers owing to recruitment being conducted in only one hospital setting. Therefore, the generalisability of these results is limited. Further research is required to evaluate the effectiveness of home-based breastfeeding peer support among women with low breastfeeding self-efficacy in a diverse population of women who are at high risk of weaning before six months postpartum.

## Conclusions

This study demonstrated some challenges to be considered in a full RCT, such as regarding the recruitment method and cultural issues with family members. In terms of potential efficacy, peer support plus usual care did not show significant differences for breastfeeding self-efficacy but were associated with a generally lower risk of weaning from exclusive breastfeeding when compared with usual care, a finding that warrants further study. Modifications to the intervention, such as to its intensity and targeting women with lower breastfeeding self-efficacy, or including telephone support and technology alongside home visits may be more appropriate in a larger, adequately powered trial. Implementing the programme early during the antenatal phase, with the consent of both parents, and tailoring the intervention towards women-centred peer support to sustain exclusive breastfeeding as well as other feeding modes, should be incorporated into a future home-based peer support arm.

## Data Availability

The datasets used and/or analysed during the current study are available from the corresponding author on reasonable request.

## Abbreviations

BSES-SF: Breastfeeding Self-Efficacy Scale Short-Form
CI: Confidence intervals
CONSORT: Consolidated Standards of Reporting Trials
NGO: Non-government organisation
RCT: Randomised controlled trials
SD: Standard deviation
WHO: World Health Organization
